# Clinical Robotic Surgery Association (India Chapter) and Indian rectal cancer expert group’s practical consensus statements for surgical management of localized and locally advanced rectal cancer

**DOI:** 10.3389/fonc.2022.1002530

**Published:** 2022-10-04

**Authors:** S. P. Somashekhar, Avanish Saklani, Jagannath Dixit, Jagdish Kothari, Sandeep Nayak, O. V. Sudheer, Surender Dabas, Jagadishwar Goud, Venkatesh Munikrishnan, Pavan Sugoor, Prasanth Penumadu, C. Ramachandra, Shilpa Mehendale, Akhil Dahiya

**Affiliations:** ^1^ Department of Surgical Oncology, Manipal Hospital, Bengaluru, Karnataka, India; ^2^ Department of Surgical Oncology, Tata Memorial Hospital, Mumbai, Maharashtra, India; ^3^ Department of GI Surgery, HCG Hospital, Bengaluru, Karnataka, India; ^4^ Department of Surgical Oncology HCG Hospital, Ahmedabad, Gujarat, India; ^5^ Department of Surgical Oncology, Fortis Hospital, Bengaluru, Karnataka, India; ^6^ Department of GI Surgery and Surgical Oncology, Amrita Institute of Medical Science, Kochi, Kerala, India; ^7^ Department of Surgical Oncology, BL Kapur-Max Superspeciality Hospital, Delhi, India; ^8^ Department of Surgical Oncology, AOI Hospital, Hyderabad, Telangana, India; ^9^ Department of Colorectal Surgery, Apollo Hospital, Chennai, Tamil Nadu, India; ^10^ Department of Surgical Oncology, Kidwai Memorial Institute of Oncology, Bengaluru, Karnataka, India; ^11^ Department of Surgical Oncology, JIPMER, Puducherry, India; ^12^ Director and Head, Department of Surgical Oncology, Kidwai Memorial Institute of Oncology, Bengaluru, Karnataka, India; ^13^ Department of Clinical and Medical Affairs, Intuitive Surgical, California, CA, United States

**Keywords:** consensus statement, rectal cancer, rectum, low-anterior resection, abdominoperineal resection, local excision, circumferential resection margin, total mesorectal excision

## Abstract

**Introduction:**

There are standard treatment guidelines for the surgical management of rectal cancer, that are advocated by recognized physician societies. But, owing to disparities in access and affordability of various treatment options, there remains an unmet need for personalizing these international guidelines to Indian settings.

**Methods:**

Clinical Robotic Surgery Association (CRSA) set up the Indian rectal cancer expert group, with a pre-defined selection criterion and comprised of the leading surgical oncologists and gastrointestinal surgeons managing rectal cancer in India. Following the constitution of the expert Group, members identified three areas of focus and 12 clinical questions. A thorough review of the literature was performed, and the evidence was graded as per the levels of evidence by Oxford Centre for Evidence-Based Medicine. The consensus was built using the modified Delphi methodology of consensus development. A consensus statement was accepted only if ≥75% of the experts were in agreement.

**Results:**

Using the results of the review of the literature and experts’ opinions; the expert group members drafted and agreed on the final consensus statements, and these were classified as “strong or weak”, based on the GRADE framework.

**Conclusion:**

The expert group adapted international guidelines for the surgical management of localized and locally advanced rectal cancer to Indian settings. It will be vital to disseminate these to the wider surgical oncologists and gastrointestinal surgeons’ community in India.

## Introduction

Colorectal cancer (CRC) is the third most common cancer globally and accounted for more than 1.9 million new cases in the year 2020 (10.0% of all cancer cases) (1). More than 60% of cases have been reported from the developed world. Within the colorectum, rectal cancer accounted for more than 730,000 new cases and an estimated 339,000 deaths in 2020 worldwide (1). In India, approximately 37,000 new rectal cancer cases (approximately 22,000 in males and approximately 15,000 in females) were reported in 2020 and the same are expected to reach over 41,500 cases by 2025 (2).

From a treatment perspective, multidisciplinary and multimodality treatment is appropriate for the management of rectal cancer (3). These consensus statements focus on the surgical management of localized and locally advanced rectal cancer; Stage I, II, and III as per the American Joint Committee on Cancer (AJCC), TNM Staging System for Rectal Cancer 8th ed. 2017. For surgical management, there are standard treatment guidelines that are advocated by recognized international bodies (3, 4). Due to the significant disparities and uniqueness of the Indian population, there are limitations and challenges to the direct application of international guidelines in the Indian context.

With these considerations, Clinical Robotic Surgery Association (India Chapter) constituted an Indian rectal cancer expert group to create uniform India-specific guidance for the surgical management of Stage I, II, and III rectal cancer.

### The expert group has used the following definitions for this project

#### Definition of rectal cancer

* Tumors within 15 cm of the anal verge by endoscopy are classified as rectal cancers for the purpose of this document (notwithstanding, the length may vary based on a number of factors)

##### Staging reference

* American Joint Committee on Cancer (AJCC), TNM Staging System for Rectal Cancer 8th ed., 2017 (5)

## Materials and methods

### Selection criteria

Selection criteria for the expert group were: (1) at least 10 years of experience as a specialist in managing rectal cancer and practicing in the public or private healthcare sector, (2) experience with radical rectal surgery approaches with both open as well as minimally invasive techniques, (3) the current location of practice at a tertiary care teaching hospital. Representation from the government and private sectors was encouraged. In addition, the emphasis was placed on equitable representation of all regions/areas of India.

### Broad question categories

Following the constitution of the expert group, the members identified three broad categories of questions: (1) locoregional and metastatic staging of rectal cancer, (2) restaging after neoadjuvant therapy, and (3) surgical management of localized and locally advanced rectal cancer. A total of twelve (12) clinical questions were defined for these 3 broad categories.

### Literature review

The expert group conducted an extensive review of the literature on randomized controlled trials, observational studies, reviews, and clinical guidelines that addressed the three broad categories of questions. A systematic search of PubMed and Embase was performed from January 1, 2010 to March 1, 2022. A total of 2125 screened articles were evaluated for their level of evidence, favoring clinical trials, meta-analysis/systematic reviews, comparative studies, and large registry retrospective studies over single institutional series, retrospective reviews, and peer-reviewed, observational studies. This evidence was graded as per the levels of evidence by the Oxford Centre for Evidence-Based Medicine (6).

### Drafting consensus statements

The expert group members drafted the consensus statements using the modified Delphi methodology (7). The members extensively discussed the available published clinical evidence and recommendations from international bodies and their real-life experiences as well as practical challenges. Drafts were circulated *via* e-mail to all the experts and multiple rounds of reviews took place. The strength of consensus statements was graded as “strong” or “weak” based on the GRADE methodology (8). The strength of each recommendation was determined by the quality of the evidence, the balance between the desirable and undesirable effects of treatment strategies, uncertainty or variability in values and preferences, and uncertainty about whether the intervention represents a rational use of resources.

### Definition of consensus

A consensus statement was accepted only if ≥75% of the experts were in agreement to the draft consensus statement. A Likert scale was used with 3 possible options: accept completely; accept with minor changes, and reject. Only those statements in which the response was “accept completely” or “accept with minor changes were accepted by the expert group.

## Results: 12 consensus statements

All the 12 consensus statements are listed in Box-1. These recommendations have been graded as strong or weak based on the GRADE framework (8). The quality of evidence has been graded as per the Oxford levels of evidence (6), in which the evidence categories included high quality, moderate quality, low quality, and very low quality.

Box 1 **Consensus statements.**

**Table:** 

**Sr. No.**	**Clinical Question**	**Consensus statement**	**Strength of recommendation and quality of evidence**
1.	What is/are the optimal radiological investigation(s) for the locoregional staging of rectal cancer?	Rectal cancer protocol (as per the Mercury study) Pelvic high-resolution MRI is the radiological investigation of choice for the locoregional staging of rectal cancer. Endorectal Ultrasound may be considered when differentiating between early T-stages (T1 *vs.* T2) or where MRI is contraindicated.	Strong recommendation, moderate quality evidence
2.	What are the optimal radiological investigations for metastatic staging in a known localized-locally advanced disease?	Chest imaging by CT scan and abdominal imaging by CT or MRI is the radiological investigation of choice for metastatic survey in a known localized/locally advanced rectal cancer case. PET CT is not routinely indicated and may be offered as an option to evaluate equivocal findings on the CT abdomen.	Strong recommendation, low-quality evidence
3.	Should patients be re-staged after neoadjuvant therapy and what is/are the optimal radiological investigation(s) for re-staging?	Yes, patients with locally advanced rectal cancer who have received neoadjuvant therapy should be re-staged. Locoregional re-staging should be done by Rectal cancer protocol (as per the Mercury study), and Pelvic high-resolution MRI. CT scan of the thorax and abdomen is not routinely recommended, and it should be offered only to patients who are poor responders to MRI restaging or for those patients who have poorly differentiated tumors.	Strong recommendation, low-quality evidence
4.	Is a multidisciplinary team needed for the management of rectal cancer?	Yes, a multidisciplinary team is needed, as it improves outcomes of the management of rectal cancer. A multidisciplinary team including members of the surgery team, medical oncologists, radiation oncologists, gastroenterologists, radiologists, genetic counselors, stoma care counselors, and other teams (like anaesthetist, dietician, rehabilitation medicine specialist), as required, should discuss the management plan.	Strong recommendation, low-quality evidence
5.	Which patients are candidates for local excision?	Local excision may be offered only to select patients with T1N0 disease who don’t have high-risk features. Selection of T1N0 patients for local excision: small (<3 cm) adenocarcinomas limited to <30% of the rectal circumference, within 8 cm from the anal verge, well or moderately differentiated, without lymphovascular invasion, perineural invasion, and tumor budding on tissue biopsy, and no clinical nodal involvement. Local excision is not an appropriate treatment option for T2 or higher lesions, with the existing evidence.	Strong recommendation, high-quality evidence
6.	What is the optimal radical resection approach for upper, middle, and lower rectal lesions in localized and locally advanced rectal cancer?	For upper third rectal cancer, tumor-specific mesorectal/partial mesorectal excision is recommended with a distal resection margin of 5 cm. The mucosal margin should be greater than the mesorectal margin. A circumferential resection margin of >2 mm is recommended. Post resection, grade, and quality of TME should be assessed. For mid and lower third rectal cancer Total Mesorectal Excision (TME) is recommended. 2 cm distal resection margin is desirous; 1 cm is acceptable and in patients who have received neoadjuvant chemoradiation sub-centimeter margin may be acceptable. The mucosal margin should be greater than the mesorectal margin. A circumferential resection margin of >2 mm is recommended. Post resection, grade, and quality of TME should be assessed.	Strong recommendation, high-quality evidence
7.	What is the optimal radical resection approach for very low-lying rectal cancer including those involving the sphincter?	In specialized centers, sphincter preservation is feasible in most cases when the rectal tumor is located 2 cm above the anorectal ring. Sphincter preservation can be carried out with acceptable anorectal function and oncologic outcome by using the technique of ultra-low anterior resection or intersphincteric resection (ISR). An APR may be prudent in patients who have infiltration of the external anal sphincter or levator ani muscles, impaired preoperative anal sphincter function, and body habitus or pelvic anatomy that makes sphincter preservation technically challenging Extralevator abdominoperineal resection (ELAPE) may be offered as an alternative to APR to carefully identified patients with documented levator involvement on MRI.	Weak recommendation, moderate quality evidence
8.	Which is the most optimal technique (open or laparoscopic or robotic-assisted) for transabdominal TME?	**8a.** Transabdominal TME for T1-T3, non-obstructing rectal cancer can be performed either by open, laparoscopic, or robotic-assisted techniques; based on access to such techniques, surgeon preference, and skills. For T4, obstructing rectal cancer; an open technique is preferred. **8b.** If expertise and technology are available; for indicated cases, minimally invasive surgery (MIS) techniques offer comparable oncological outcomes as compared to open techniques. MIS techniques, particularly robotic-assisted surgery, also offer a better field of vision. **8c**. Of the two MIS techniques, the robotic-assisted technique offers superiority over laparoscopic surgery in patients with high BMI, male pelvis, and where sphincter preservation is contemplated. **8d.** A multicentric study from India (prospective in nature, if feasible) is needed to assess the perioperative, functional, and oncological outcomes of open, laparoscopic, and robotic-assisted surgery for radical rectal resection.	Strong recommendation, moderate-quality evidence
9.	For radical rectal resections, where should the inferior mesenteric artery be ligated (high tie or low tie)?	In the majority of cases, a low tie is appropriate. In select cases, when clinically suspicious lymph nodes are present at the level of the inferior mesenteric artery, a high tie is indicated.	Strong recommendation, moderate-quality evidence
10.	What is the method of choice in assessing anastomotic perfusion in radical rectal resection surgery with anastomosis?	Clinical assessment to check anastomotic integrity should be done routinely. This may be supplemented with an assessment of perfusion by indocyanine green dye.	Strong recommendation, low-quality evidence for dye
11.	What is the current status of Transanal total mesorectal excision (t-TME) in radical rectal surgery in Indian settings?	t-TME cannot be recommended in Indian settings with the existing evidence. There is a need for feasibility studies in Indian settings followed by comparative studies.	Strong recommendation, moderate quality evidence
12.	Is the wait-and-watch approach appropriate for low-lying rectal cancer patients who are in clinical complete response to neoadjuvant therapy?	Wait and watch approach after clinical complete response to neoadjuvant therapy cannot be advocated for routine practice with the existing evidence. This approach may be offered as an alternate option to patients who require permanent stoma or are very frail. These patients should have a clinically complete response on rectal examination, endoscopy, and MRI. A multidisciplinary team should ensure complete monitoring of these patients. Patients need to be adequately counseled for strict adherence to strict follow-ups. A special consent form needs to be devised to avoid future litigation.	Strong recommendation, moderate-quality evidence

**Figure 1:**
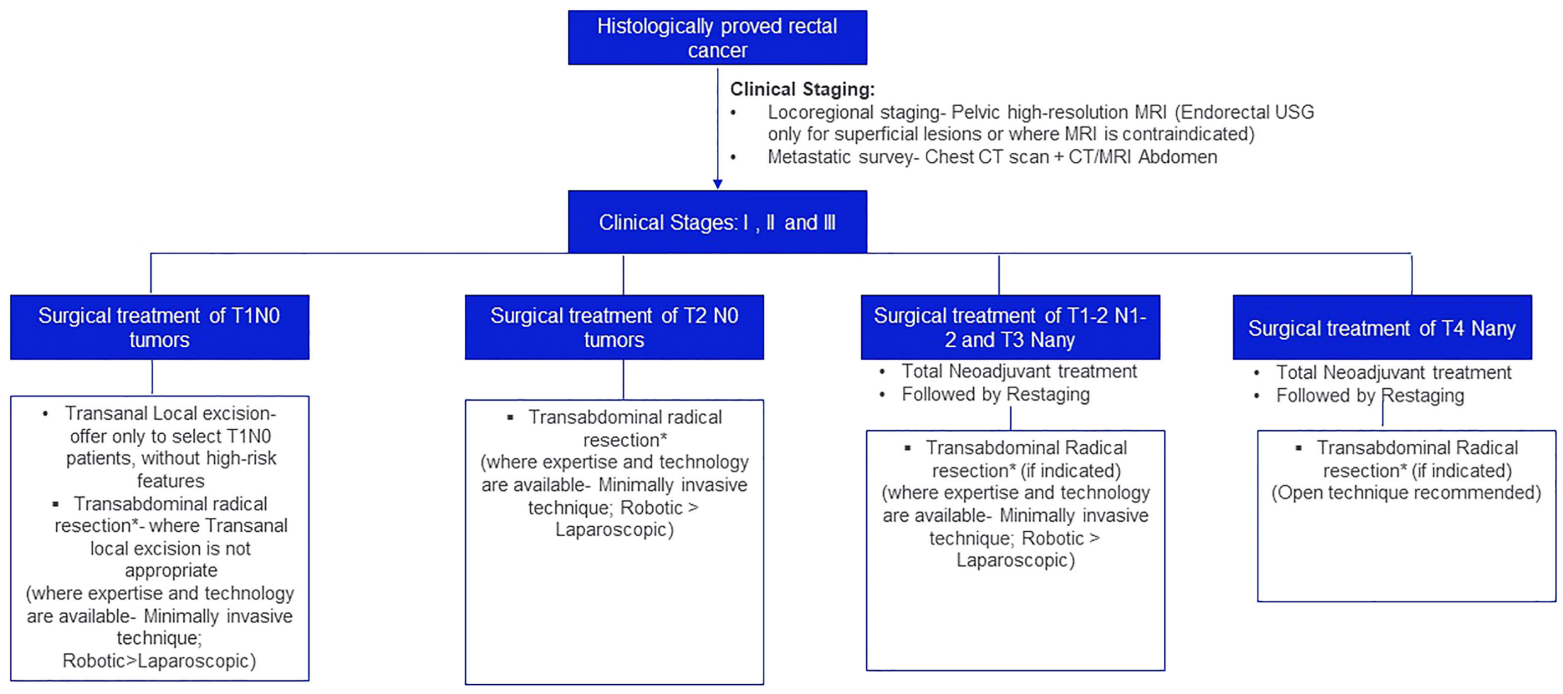
Treatment algorithm for the surgical management of localized and locally advanced rectal cancer. *Principles of Transabdominal radical resection: For third rectal cancer, tumor-specific mesorectal/partial mesorectal margin. A circumferential resection margin of >2 mm is recommended. For mid and lower third rectal cancer, Total Mesorectal Excision (TME) is recommended. 2 cm distal resection margin is desirous; 1 cm is acceptable and in patients who have recived neoadjuvant chemoradiation sub-centimeter margin may be acceptable. The mucosal margin should be greater than the mesorectal margin. A circumferential resection margin of >2mm is recommended.

The treatment algorithm for the surgical management of localized and locally advanced rectal cancer is depicted in **Figure 1**.

## Discussion

There is no unified guidance document from India which provides specific guidance for the surgical management of rectal cancer. As a result of disparities in access and affordability of various treatment options, there is an unmet need to personalize the standard treatment guidelines advocated by the recognized international physician societies in the Indian context. The expert group set up by the CRSA (India) has customized international guidelines for the surgical management of localized and locally advanced rectal cancer in Indian settings. The final consensus statements on the three broad areas of focus covering 12 clinical questions are summarized below.

### Broad category: Staging and re-staging

#### Q1: What is/are the optimal radiological investigation(s) for the locoregional staging of rectal cancer?

Consensus statement: *Rectal cancer protocol (as per the Mercury study). Pelvic high-resolution MRI is the radiological investigation of choice for the locoregional staging of rectal cancer. Endorectal Ultrasound may be considered when differentiating between early T-stages (T1 vs. T2) or where MRI is contraindicated.*

*Strong recommendation, moderate-quality evidence*

Pelvic MRI helps to assess the depth of tumor penetration and the presence/absence of lymph node involvement. Pelvic MRI helps to predict the circumferential resection margin (CRM) pre-operatively and can differentiate low-risk patients from high-risk pre-operatively. This has clear implications for disease-free survival and the overall survival of the patients. The Panel recommends that the Pelvic high-resolution MRI must report the tumor length, circumferential location of the tumor, T-stage, Nodal stage, deposits within the mesorectum, Involvement of the mesorectal fascia, suspicious extramesorectal lymph nodes, the extent (mm) of extramural growth or depth of invasion.

The primary evidence for pelvic MRI emanates from the Mercury study, which after a 5-year follow up concluded that high-resolution MRI helps predict CRM pre-operatively and predicts the risk of local recurrence and distant metastasis (9). The prospective, multi-center, Mercury II study concurs with the findings of the Mercury study, particularly in the assessment of the low rectal plane and prediction of CRM in this setting (10). Similarly, other retrospective studies have similar results and establish the role of pelvic MRI in optimizing the treatment of these patients (11, 12).

CT scan for the purpose of loco-regional staging has limited evidence and that too doesn’t support its use over pelvic MRI and it is not recommended by standard guidelines (3, 13). Endoscopic ultrasound (EUS) has shown disappointing results in the T-staging of rectal cancer (3). In a UK study by Ashraf and colleagues, EUS inaccurately staged rectal cancer in 44.8% of tumors: 32.7% were under-staged and 12.1% were over-staged (14). Further, operator dependence and EUS’ inability to fully image high or bulky tumors limit its utility in clinical practice (15, 16). However, this panel believes EUS may have a role in very select settings when differentiating between early T-stages (T1 *vs.* T2) or where MRI is contraindicated. We could not find any Indian study (prospective or retrospective) on this topic.

#### Q2: What are the optimal radiological investigations for metastatic staging in known localized-locally advanced disease?

*Consensus statement: Chest imaging by CT scan and abdominal imaging by CT or MRI is the radiological investigation of choice for metastatic survey in a known localized/locally advanced rectal cancer case. PET CT is not routinely indicated and may be offered as an option to evaluate equivocal findings on the CT abdomen.*

*Strong recommendation, low-quality evidence*

There is limited prospective evidence from imaging meant for distant staging. A Korean study by Choi and colleagues showed the superiority of CT chest over Chest X-Ray for pre-operative detection of lung metastases in rectal cancer (17). The evidence for CT or MRI abdomen is very limited and has primarily emanated from expert opinions (Level 5) (3).

The routine use of PET scan for metastatic imaging is not supported by current evidence, which is very limited. A prospective, Spanish study by Ramos and colleagues concluded that PET had limited utility in staging liver metastases in rectal cancer (18). Usually, PET scan uptake in non-mucinous tumors is good. In Mucinous tumors, its utility is very limited and hence Pelvic MRI + CECT scan abdomen and thorax is a better tool for these patients.

We could not find any Indian study for evaluation of CT chest or CT/MRI abdomen for metastatic staging. A prospective, single, arm from TATA, Mumbai evaluated the role of PET CT in detecting systemic metastatic spread in rectal cancers with lateral pelvic lymph nodes. This study reported that the addition of PET CT to other imaging modalities led to the detection of additional extra-pelvic metastasis in more than 11% of patients (19). PET CT needs to be used judiciously in carefully selected patients and is not routinely indicated (3).

#### Q3: Should patients be re-staged after neoadjuvant therapy and what are the optimal radiological investigations for re-staging?

*Consensus statement: Yes, patients with locally advanced rectal cancer who have received neoadjuvant therapy should be re-staged. Locoregional re-staging should be done by Rectal cancer protocol (as per the Mercury study) and Pelvic high-resolution MRI. CT scan of the thorax and abdomen is not routinely recommended, and it should be offered only to patients who are poor responders to MRI restaging or for those patients who have poorly differentiated tumors.*

*Strong recommendation, low-quality recommendation*

In patients who have undergone neoadjuvant treatment followed by radical resection, there are tumor regression grading systems available- like AJCC/CAP tumor regression grading system, Mandard system, etc. These systems may help guide the management of adjuvant therapy and impact the long-term survival of patients. However, in patients who have only undergone neoadjuvant treatment and not radical resection- accurate estimation of pCR is a challenge. In the absence of a histological specimen, imaging plays a key role. Endoscopic assessment by Endorectal Ultrasound with cross sectional imaging by pelvic MRI and CT scan of thorax/abdomen help identify patients who are in partial or complete clinical response (20). There is limited prospective evidence from imaging meant for re-staging. Most of the global evidence is retrospective in nature. EUS alone for re-staging has yielded disappointing results in multiple small retrospective studies (challenges include unable to detect tumor foci in patients with normal EUS and unable to detect lymph node involvement) (21, 22). Hence, imaging by pelvic MRI and CT have largely supplanted its use. A retrospective Indian study concluded that unsafe-MRI assessed CRM in an MRI after neoadjuvant therapy was significantly associated with pathological CRM (23). Another retrospective study by the same group at TATA, Mumbai evaluated the accuracy of pelvic MRI for nodal re-staging in 166 locally advanced rectal cancer cases who have undergone neoadjuvant therapy. The study reported a satisfactory negative predictive value, but the positive predictive value was poor, and the accuracy was moderate (24).

For CT abdomen and thorax in re-staging settings, relatively recent retrospective data have shown that these investigations are low-yield in these settings and may not significantly alter the management plan (25, 26). For Indian settings, a 2016 study from TATA Mumbai reported that tumor grade was the most important predictor of disease progression and patients with high grade, poorly differentiated tumors benefit the most from re-staging (27). In the absence of good quality evidence, expert opinions have largely provided the only evidence. At this time, the expert opinions favor the use of the same radiological investigations as were used for initial staging (3).

### Broad category: Surgical management of localized and locally advanced rectal cancer

#### Q4: Is a multidisciplinary team needed for the management of rectal cancer?

*Consensus statement: Yes, a multidisciplinary team is needed, as it improves outcomes of management of rectal cancer. A multidisciplinary team including members of the surgery team, medical oncologists, radiation oncologists, gastroenterologists, radiologists, genetic counselors, stoma care counselors, and other teams (like anaesthetist, dietician, rehabilitation medicine specialist), as required, should discuss the management plan.*

*Strong recommendation, low-quality evidence*

The recommendation is based primarily on expert opinions (Level 5) and at this time there is no prospective evidence to support its impact on patient outcomes (3).

#### Q5: Which patients are candidates for local excision?

*Consensus statement: Local excision may be offered only to select patients with T1N0 disease who don’t have high-risk features. Selection of T1N0 patients for local excision: small (<3 cm) adenocarcinomas limited to <30% of the rectal circumference, within 8 cm from the anal verge, well or moderately differentiated, without lymphovascular invasion, perineural invasion, and tumor budding on tissue biopsy, and no clinical nodal involvement.*

*Local excision is not an appropriate treatment option for T2 or higher lesions, with the existing evidence.*

*Strong recommendation, moderate-quality evidence*

Local resection is indicated only for a select group of patients with T1N0 disease (3). Amongst local excision techniques for rectal cancer, conventional transanal local excision has given way to transanal endoscopic microsurgery (TEMS) and minimally invasive techniques, primarily due to better visualization and access to proximal tumors (28, 29). The DUTCH trial by de Graaf and colleagues compared TEMS to radical rectal surgery in T1N0 tumors and reported significantly higher local recurrence rates for TEMS (24% *vs.* 0%), although they did show improvement in some perioperative outcomes like blood loss and length of hospital stay (30). Several other observational studies have reported a substantially increased risk of local recurrence with TEMS, necessitating salvage abdominoperineal resection, and pelvic exenteration in some cases, leading to compromised clinical outcomes (31–33).

There is emerging evidence for the role of TEMS after neoadjuvant chemoradiotherapy in T1N0 as well as T2N0 tumors. An Italian group in 2006 published findings of their randomized controlled trial evaluating TEMS *vs.* laparoscopic resection following neoadjuvant therapy in T2N0 low rectal cancers and reported comparable local recurrence rates and survival at a follow-up of 3 years (34) and their findings were similar when they published an update with minimum 5-years follow up (35). The multicentric CARTS study evaluated long-term oncological outcomes and health-related quality of life (HRQL) in patients with cT1-3N0M0 rectal cancer who underwent neoadjuvant chemoradiotherapy (CRT) followed by TEMS. Two-thirds of patients underwent TEMS after CRT and reported acceptable oncological outcomes and quality of life, but 22-50% of patients reported varying degrees of bowel dysfunction. Also, one-third had to undergo radical surgery (36). A 2021 metanalysis of randomized controlled trials of TEMS *vs.* radical surgery concludes that in T1N0 patients may be offered TEMS, but in locally advanced cases radical surgery remains the mainstay (37).

An additional approach been evaluated in clinical trials aims at comparing TEMS to radical surgery after patients have received a short course of radiotherapy. The 2021 UK TREC randomized controlled trial with 55 patients reported significantly higher levels of organ preservation with TEMS with lower morbidity and a trend towards improved quality of life (38). A larger STAR-TREC randomized controlled trial is further studying oncological outcomes with this approach.

Specifically, for India, the authors could find only one study from India, which had evaluated local excision in early-stage rectal cancer. This prospective study enrolled 36 benign rectal polyps and 12 malignant rectal cancer cases and didn’t report any local recurrence in malignant cases with a follow-up ranging from 1 to 3 years (39).

#### Q6: What is the optimal radical resection approach for upper, middle, and lower rectal lesions in localized and locally advanced rectal cancer?

*Consensus statement: For upper third rectal cancer, tumor-specific mesorectal/partial mesorectal excision is recommended with a distal resection margin of 5 cm. The mucosal margin should be greater than the mesorectal margin. A circumferential resection margin of >2 mm is recommended. Post resection, grade, and quality of TME should be assessed.*

*For mid and lower third rectal cancer Total Mesorectal Excision (TME) is recommended. 2 cm distal resection margin is desirous, 1 cm is acceptable, and in patients who have received neoadjuvant chemoradiation sub-centimeter margin may be acceptable. The mucosal margin should be greater than the mesorectal margin. A circumferential resection margin of >2 mm is recommended. Post resection, grade, and quality of TME should be assessed.*

*Strong recommendation, high-quality evidence*

The question of the plane of resection in radical rectal surgery has been primarily addressed by two prospective studies, one from the UK (MRC CR07-NCIC-CTG CO16) and the second one from the Germany (CAO/ARO/AIO-04, a Phase 3 randomized controlled trial) (40, 41). Both the studies concluded the plane of resection (mesorectal *vs.* muscularis propria) is an independent predictor of local recurrence and the mesorectal plane has lower local recurrence rates as well as improved survival.

A tumor-specific mesorectal excision with a distal 5-cm mesorectal resection margin is sufficient for upper rectal cancer. Studies that have examined the mesorectum of resected upper rectal cancer have shown that lymph node metastasis in the mesorectum is rare beyond 5 cm distal to the mucosal edge of the tumor (42, 43). Mesorectal excision beyond 5 cm distal to the tumor may result in an increased risk of anastomotic leakage without any oncologic benefit (44). With respect to the length of the distal resection margin, the practice has evolved over the years. A distal margin of 2 cm or more was considered adequate in the 1980s, which decreased to 1cm and now even a sub-centimeter distal resection margin after pre-operative neoadjuvant chemoradiation is considered to offer acceptable oncological outcomes (45). A 2019 study from TATA Mumbai concluded that for middle and low rectal cancers, the overall, local, and systemic recurrence rates were found to be similar in all distal resection margin groups (6 mm, 6-10 mm, >10 mm) (45).

The decision of offering low anterior resection (LAR) for upper rectal cancer is very established and the panel has chosen not to delve into this question. A prospective case series by Enker et al. in 1999 was one of the largest to report perioperative and long-term outcomes of LAR in localized and locally advanced rectal cancer (46). This study analyzed 681 consecutive LAR cases, more than 58% of these were in the middle rectum, approximately 21% were upper rectal cases and the rest were low rectal cases. The study reported a 5-year overall survival of >80% for LAR, with a local recurrence rate of 10% (46).

For middle and lower rectal cancers, evidence for comparison of LAR *vs.* abdominoperineal resection (APR) is lacking. The majority of the existing evidence has emanated from prospective non-comparative studies or retrospective comparative studies. A 2013 Korean study retrospectively compared APR (n=402) to LAR (n=402) for lower rectal cancers and reported CRM positivity to be a more frequent risk with APR (1.6-fold) and was significantly associated with local and systemic recurrence (47). The Swedish cancer registry trial analyzed >13,000 patients, more than 50% of these were LARs and approximately 25% were APRs. The 5-year relative survival rate for LAR was 70%, whereas it was approximately 60% for APR (48). In addition, a pooled analysis of 5 European trials with more than 3500 APR cases reported that APR, as a procedure itself, is associated with an increased risk of recurrence and death (49). A systematic review by How and colleagues reported significantly lower recurrence rates and better survival with LAR as compared to APR (50). In terms of quality of life, prospective and retrospective comparative studies have reported improved outcomes with LAR as compared to the APR (51, 52). The NSABP R-04 randomized controlled trial published the patient-reported outcomes for APR as compared to the LAR. Sexual and micturition-related symptoms were significantly worse in the APR group (52).

#### Q7: What is the optimal radical resection approach for very low-lying rectal cancer including those involving the sphincter?

*Consensus statement: In specialized centers, sphincter preservation is feasible in most cases when the rectal tumor is located 2 cm above the anorectal ring. Sphincter preservation can be carried out with acceptable anorectal function and oncologic outcome by using the technique of ultra-low anterior resection or intersphincteric resection (ISR). An APR may be prudent in patients who have infiltration of the external anal sphincter or levator ani muscles, impaired preoperative anal sphincter function, and body habitus or pelvic anatomy that makes sphincter preservation technically challenging. In spite of some publications observing a possible benefit in terms of reduction in CRM involvement, iatrogenic perforations, or local recurrence, there is not enough evidence to affirm the superiority of ELAPE compared to conventional APE in terms of oncological results. Extralevator abdominoperineal resection (ELAPE) may be offered as an alternative to APR to carefully identified patients with documented levator involvement on MRI.*

*Weak recommendation, moderate-quality evidence*

Sphincter-preserving resection as the procedure of choice in rectal cancer has been validated by several studies. Although there are no randomized trials, many comparative studies have reported that sphincter preservation provides similar short- and long-term oncologic outcomes compared with abdominoperineal resection (53, 54). In addition, quality of life may be significantly more improved with sphincter preservation than with APR, although the anorectal function is not always perfect in patients treated with sphincter-preserving procedures (55).

The ultralow anterior resection removes the rectum en bloc near the attachment point at the puborectalis for tumors located 1 to 2 cm above the dentate line (56). For ultralow rectal cancer, the absence of the mesorectum in the most distal portion is the key to the interest in removing the internal sphincter to widen the CRM unless the tumor has invaded the external sphincter. ISR facilitates the achievement of a negative distal resection margin by transanal division and resection of all or part of the internal anal sphincter (57). Rullier et al. analyzed the oncologic outcome of ISR in tumors located between 1.5 and 4.5 cm from the anal verge and reported that complete microscopic resection was possible in 89% of the cases, with a local recurrence rate of 2% (58). The incidence of major fecal incontinence is higher in the ISR group with similar overall survival and disease-free survival rates when compared to ULAR (59, 60).

ELAPE technique involves a wider tissue removal and, as some studies have shown, also a reduction in CRM involvement or intraoperative perforation (61). Chen et al. describe the lower intraoperative perforation rate of ELAPE than APE (RR =0.52, P=0.002), without significant differences of CRM involvement (RR =0.72, P=0.10) and local recurrence rate [(odds ratio (OR) =0.55, P=0.17)] (62). Negoi et al. describe similar results concluding that ELAPE significantly lessens the intraoperative perforation incidence, with no benefits in regard to CRM infiltration and local recurrence rate (63). The Spanish study did not find differences between APE and ELAPE in terms of CRM involvement (13.1% *vs.* 13.6%; P=0.846), intraoperative tumor perforation (7.9% *vs*. 7.7%; P=0.902) and local recurrence rate at 2 years (2.7% *vs*. 5.6%; P=0.664) (64). Similarly, Zhou et al. published a meta-analysis in 2015 and did not find differences in those criteria either (65).

Nevertheless, other authors showed the lower intraoperative perforation rate and local recurrence in ELAPE compared to conventional APE, with greater CRM involvement in the conventional APE group without statistical significance (66). Moreover, overall survival and progression-free survival were similar between groups, even after that survival was analyzed according to TNM stage, T stage, N stage, and with or without neoadjuvant chemoradiotherapy.

ELAPE requires the removal of more perirectal tissue and may increase the chance of injury to the pelvic and perineal nerves, which may increase the occurrence of postoperative complications such as sexual dysfunction, urinary retention, and chronic perineal pain. Based on the study of pelvic anatomy and postoperative complications, Han et al. considered that it is not necessary to remove the entire levator ani muscle if a tumor is limited to one sidewall, or the tumor is staged as T3 (67). This requires the assurance of preoperative magnetic resonance imaging (MRI) evaluation of rectal cancer and the extent of tumor invasion to the rectal wall. The concept of individualized ELAPE surgery endorses the extent of surgical resection as determined by precise preoperative MRI imaging (68).

#### Q8: Which is the most optimal technique (open or laparoscopic or robotic-assisted) for transabdominal TME?

*Consensus statement: (a) Transabdominal TME for T1-T3, non-obstructing rectal cancer can be performed either by open, laparoscopic, or robotic-assisted techniques; based on access to such techniques, surgeon preference, and skills. For T4, obstructing rectal cancer; an open technique is preferred. (b) If expertise and technology are available; for indicated cases, minimally invasive surgery (MIS) techniques offer comparable oncological outcomes as compared to open techniques. MIS techniques, particularly robotic-assisted surgery, also offer a better field of vision. (c) Of the two MIS techniques, the robotic-assisted technique offers superiority over laparoscopic surgery in patients with high BMI, male pelvis, and where sphincter preservation is contemplated. (d) A multicentric study from India (prospective in nature, if feasible) is needed to analyze the perioperative, functional, and oncological outcomes of open, laparoscopic, and robotic-assisted surgery for radical rectal resection.*

*Strong recommendation, moderate-quality evidence*

The authors could find only two studies where the three approaches (open, laparoscopic, robotic-assisted) have been evaluated together in a single study. A 2016 single-centric retrospective comparison of 300 patients, across the three techniques, reported similar CRM involvement and lymph node harvest (69). A 2020 single-centric Indian study that analyzed a total of 100 cases (25 open, 25 laparoscopic, and 50 robotic) showed robotic-assisted surgery had a trend towards improved TME completeness and CRM (70).

When reviewing studies that have evaluated two approaches, some studies have found the laparoscopic approach to be comparable to the open approach in terms of short and long-term outcomes (Color II study group and COREAN trial) (71, 72). Whereas in other studies, the laparoscopic approach has reported inferior outcomes in terms of TME completeness and CRM (ACOSOG Z6051 RCT and ALaCaRT RCT) (73, 74). There are limited prospective studies that have compared open to robotic-assisted techniques. The highest level of evidence comes from an Indian RCT in 2015 with 50 cases, 25 each of open and robotic-assisted approaches. The study reported shorter hospital stay, zero conversion rate, and higher lymph node yield with the robotic approach (75). In real-world evidence studies, robotic-assisted surgery *vs.* open has demonstrated longer surgery times, but shorter hospital stays as well as lower blood loss and comparable rates of anastomotic leaks (76–78).

Studies evaluating robotic-assisted surgery versus laparoscopic surgery have also reported mixed results, favoring robotic or showing comparable outcomes. The Robotic *vs*. Laparoscopic Resection for Rectal Cancer (ROLARR) trial, with the primary endpoint of conversion rate, did not identify any significant difference between robotic arm versus laparoscopic arm, whereas a subgroup analysis did indicate an advantage of robotic surgery in males (79). However, an analysis of the impact of the learning curve reported that the majority of surgeons in the ROLARR trial were experts in laparoscopic surgery whereas those in the robotic arm were still in their learning curve (80). The real-world evidence is considered more representative of routine clinical practice, without bias of learning curve in patient inclusion. A propensity-matched analysis of the National Clinical Database in Japan included more than 2800 patients and reported a significantly lower conversion rate in the robotic arm as compared to the laparoscopic arm (81). Similarly, a meta-analysis of >19,700 patients reported a significantly lower conversion rate with the robotic approach (82). Specifically, in Indian settings, a propensity score-matched analysis showed similar peri-operative outcomes, but significantly lower morbidity with the robotic-assisted approach (83). A meta-analysis and systematic review by Milone and colleagues in 2022 included 70 studies from different surgical specialties with 14,329 procedures (6472 robotic and 7857 laparoscopic). The robotic approach was associated with a reduced risk of conversion (OR 1.53, 95% CI 1.12-2.10, p = 0.007). The analysis of the procedures performed by “expert surgeons” showed a statistically significant difference in favor of robotic surgery (OR 1.48, 95% CI 1.03-2.12, p = 0.03). A reduced conversion rate due to adhesions with the robotic approach was observed in patients undergoing colorectal cancer surgery (OR 2.62, 95% CI 1.20-5.72, p = 0.02) (84).

#### Q9: For radical rectal resections, where should the inferior mesenteric artery be ligated (high tie or low tie)?

*Consensus statement: In the majority of cases, the low tie is appropriate. In select cases where clinically suspicious lymph nodes are present at the level of the inferior mesenteric artery, a high tie is indicated.*

*Strength of recommendation: Strong recommendation, moderate-quality evidence*

The high tie *vs*. low tie RCT provides level 1 evidence for this question, it analyzed 215 patients. The long-term survival results, 5-year disease-free survival rate, and 5-year overall survival, did not differ between the two groups (85). Similar findings were recorded in a 2020 meta-analysis of RCTs (86). In terms of functional outcomes, a 2021 meta-analysis showed significantly improved genitourinary and bowel symptoms with a low tie (87).

#### Q10: What is the method of choice in assessing anastomotic perfusion in radical rectal resection surgery with anastomosis?

*Consensus statement: Clinical assessment to check anastomotic integrity should be done routinely. This may be supplemented with an assessment of perfusion by indocyanine green dye*

*Strength of recommendation: Strong recommendation, low-quality evidence*

The only prospective evidence for use of indocyanine green (ICG) dye comes from a single-center Indian study in 2021 (88). Assessment of perfusion by ICG dye led to changes in the decision of the surgical team in more than 85% of cases.

#### Q11: What is the current status of Transanal total mesorectal excision (t-TME) in radical rectal surgery in Indian settings?

*Consensus statement: t-TME cannot be recommended as a routine practice in Indian settings with the existing evidence. There is a need for feasibility studies in Indian settings followed by comparative studies.*

*Strong recommendation, moderate quality evidence*

Transanal TME (t-TME) was introduced as an alternate approach for distal rectal tumors and preliminary studies have hypothesized that t-TME may aid better dissection of the mesorectal fascia plane in low rectal tumors, especially in obese patients and in a narrow, irradiated pelvis (89–92). Denost et al. reported that under direct vision t-TME is beneficial for margin status (93), similarly, other small retrospective studies in the early and middle 2010s reported improved quality of operative specimens and reduced CRM involvement as compared to laparoscopic transabdominal TME (89–92).

Multiple, small retrospective studies have evaluated the learning curve of t-TME. A single-centric Polish study in 2020 reported a learning curve of 40 cases to achieve t-TME proficiency (94). Intra-operative adverse events like a purse string failure, gas embolism, and bowel wall perforation have been reported, the Polish group observed stabilization of these intra-operative adverse events around the 35th case (94). Koedam et al. in a single center analysis of 138 t-TME cases, also reported a learning curve of 40 cases (95) whereas a group from Florida in 2020 noted it to be 45-51 cases (96).

In the period between 2017-early 2022, the authors note a significant spurt in a number of comparative (RCTs/prospective/retrospective) studies for t-TME. Our analysis indicates more than 25% of comparative studies (evaluating various surgical approaches in localized and locally advanced rectal cancer) were dedicated to t-TME. The multicentric RCT evidence is certainly lacking for t-TME. To bridge this data gap, multicentric COLOR III RCT and GREECAR RCT comparing transanal TME to laparoscopic TME for mid and low rectal cancer is ongoing and expected to report their preliminary results soon (97). A Chinese meta-analysis found that t-TME was associated with a lower conversion rate and shorter operative time with no difference in the rate with post-op complications, the quality of life including anal function when compared to laparoscopic arm (98). Long-term results of the Bordeaux RCT that randomized 100 patients to either t-TME or laparoscopic dissection and noted lower CRM positivity with the t-TME approach, but did not report any difference in the 5-year local recurrence rate (3% with t-TME *vs.* 5%, p 0.3) (93). An RCT from China evaluated pathological outcomes after t-TME (n=128) versus laparoscopic TME (n=133) and did not report any difference, a positive CRM was detected in 2 cases in each group (99). A prospective, non-randomized, comparative analysis of 58 cases from two centers reported comparable oncological outcomes between t-TME and laparoscopic conventional TME (100). The largest single-arm study to date includes results from the international t-TME registry, which noted t-TME to be an oncologically safe and effective technique that has acceptable short-term clinical outcomes (101).

Several retrospective studies comparing robotic surgery and t-TME for rectal cancer have revealed that they are equivalent per short-term outcomes and/or histopathological outcomes (102, 103). According to the study by Lee et al. comparing the short-term postoperative outcomes and pathological outcomes of robotic and t-TME for mid- and low-rectal cancer were closely comparable (102). The distal margin tumor involvement was observed more frequently in the t-TME group (1.8% *vs*. 0.3%; P = 0.051) as opposed to the robotic group, despite the longer length to the distal margin (16.9 mm *vs*. 15.1 mm; P = 0.097). The significance of the transanal approach will endure, particularly in cases with difficulty *via* the transabdominal approach, whether open, laparoscopic, or robotic (104). Recently, transanal use of robotic platforms has been reported to reduce the limitations of the ergonomics of single-port surgery (105–108). Furthermore, with the advent of robotic platforms designed for single-port surgery, robotic transanal surgery has been expected to overcome the limitations of single-port surgery. While these approaches might not be mutually exclusive, a combination of the modalities might lead to better outcomes, including NOTES. Although not directly related to TME, the importance of the transanal endoscopic approach might become more prominent in the near future. With the widespread adoption of the watch-and-wait strategy in the treatment of rectal cancer (109), local excision following chemoradiotherapy (CRT) to remove residual tumors and evaluate the effect of CRT has gained ground (110). The role of local excision will thus be more important in the treatment of rectal cancer and this approach will become an essential procedure for colorectal surgeons.

Considering the existing evidence, guidelines and consensus statements have advocated the use of t-TME in highly selected cases. The 2018 St. Gallen consensus on t-TME carefully identifies patients for t-TME and advocates a minimum learning curve of 20 cases performed within about 2 years (111). The 2019 American Society of Colon and Rectal Surgeons Clinical Practice Guidelines for rectal Cancer does not recommend the use of t-TME in routine practice due to a lack of established oncological outcomes and long-term follow-up (4).

Norwegian Colorectal Cancer Registry recently published t-TME outcomes in the British Journal of Surgery with the unexpectedly high rate of early multifocal local pelvic recurrence which has led to a national moratorium on t-TME for rectal cancer in Norway (112). Also, there was a high incidence of urethral injuries occurring during both the learning curve and in established practice, the unexpected incidence of carbon dioxide embolism, and high rates of morbidity during the learning curve, even within a structured national training program (112). Given the concerns raised, and while awaiting the results of the COLOR III trial, the Association of Coloproctology of Great Britain and Ireland (ACPGBI) have notified NICE of the safety concerns of t-TME. Pending further guidance, the ACPGBI and Getting It Right First Time (GIRFT) are recommending a considered pause for re-evaluation and consolidation of evidence of the t-TME approach to resecting rectal cancer (113). The ACPGBI Executive accepts that some of these recommendations are based on pragmatic common sense rather than hard evidence, especially as the learning curve for the safe independent practice of t-TME has yet to be established. The 2022 rapid guidelines by United European Gastroenterology and the European Association for Endoscopic Surgery state, with the existing evidence, t-TME cannot be recommended over laparoscopic or robotic TME for low rectal cancer (114).

#### Q12: Is the wait-and-watch approach appropriate for low-lying rectal cancer patients who are in clinical complete response to neoadjuvant therapy?

*Consensus statement: Wait and watch approach after clinical complete response to neoadjuvant therapy cannot be advocated for routine practice with the existing evidence. This approach may be offered as an alternate option to patients who require permanent stoma or are very frail. These patients should have a clinically complete response after rectal examination, endoscopy, and MRI. A multidisciplinary team should ensure complete monitoring of these patients. Patients need to be adequately counseled for strict adherence to strict follow-ups. A special consent form needs to be devised to avoid future litigation.*

*Strong recommendation, moderate-quality evidence*

The question of the “wait-and-watch” approach is only relevant for patients who have a clinical complete response, cCR, to neoadjuvant treatment for localized and locally advanced rectal cancer.

There is no level 1 evidence (RCT) or large non-randomized comparative studies for delaying radical resection in these sets of patients. The International Watch & Wait Database (IWWD) presents the largest case series of patients treated with the wait and watch approach, it included data from 47 institutes across 15 countries (109). Patients with rectal cancer in whom the standard of care, TME surgery, was omitted after neoadjuvant therapy was included. For identification of a cCR after neoadjuvant therapy, endoscopy was performed in 88·5% of cases. Chemoradiation was the most commonly used neoadjuvant therapy (91.4%). In 2018, van der Valk and colleagues presented an analysis of 880 patients from this registry. After a mean follow-up of 3.3 years, 25.3% of patients developed a local regrowth in the first 2 years of follow up and the regrowth was located in the bowel wall in 97% of cases. The 5-year overall survival was reported to be 85% and disease-specific survival was 94% (106). A 2017 meta-analysis by Dossa et al. of observational studies revealed a pooled lower local recurrence rate of 15.7% as compared to the IWWD registry (115). This group reported a comparable overall recurrence rate in the wait and watch group versus the surgery group, but the disease-free survival was significantly better in the surgery group (HR 0·47, 95% CI 0·28-0·78).

A Danish, single-centric, prospective, single-arm study evaluated the wait and watch approach specifically for low rectal cancers, T2 or T3, N0-N1 adenocarcinoma in the lower 6 cm of the rectum (116). At a median follow-up of 23.9 months, the local recurrence rate at 1 year was 15·5%. A propensity-score matched cohort analysis by a UK group reported an analysis of 129 patients managed by the wait-and-watch approach and reported a local regrowth rate of 34% at a follow-up of 33 months (117). This study reported a comparable 3-year survival rate between the wait and watch group and the surgery group.

From an Indian standpoint, a single-centric, retrospective study from TATA, Mumbai evaluated the wait-and-watch approach in patients (n=36) who had a near-complete CR (nCR) or cCR after neoadjuvant chemoradiation for low-lying rectal cancers (118). The local regrowth rate was reported to be 17% at a median follow-up of 35 months. The same group also evaluated a related question on the impact of delaying surgery (>12 weeks) after chemoradiation in a retrospective analysis of 161 patients (119). Delaying surgery by >12 weeks led to more blood loss and significantly less sphincter preservation, but the oncological outcomes were very similar to those patients who underwent surgery <12 weeks after neoadjuvant treatment (119). International guidelines have not addressed this issue and there is no clear guidance on the selection of patients for the wait-and-watch approach.

## Conclusion

The expert group has created 12 consensus statements, to be used as a guidance document, for the benefit of young surgeons who are/will be managing rectal cancer in Indian settings. The majority of questions are related to routine clinical practice and the expert panel has tried to create clear guidance for our young peers. Consensus statement 8 specifically calls out trained and experienced surgeons to collaborate and generate multicentric clinical evidence and create a database for clinical outcomes related to radical rectal surgery. Similarly, for transanal-TME in Indian settings, the group has created recommendations for the training of surgeons in this new technique.

It will be vital to disseminate these to community surgical oncologists and encourage in-clinic application. The expert panel aims to reconvene and update these guidelines once local clinical evidence is available.

## Data availability statement

The original contributions presented in the study are included in the article/supplementary material. Further inquiries can be directed to the corresponding author.

## Author contributions

All authors contributed to the study conception, design and analysis of data. All authors critically reviewed all manuscript drafts and provided comments. All authors gave their approval for the final version to be published. SP is the guarantor of this work and as such takes full responsibility for the integrity of the data and the accuracy of the data analysis.

## Funding

This work was supported by the grants from Intuitive Surgical, California, US.

## Acknowledgments

We thank Catalyst Clinical Services Pvt. Ltd. for its editorial assistance during the submission of this manuscript.

## Conflict of interest

AD and SM are employees of Intuitive Surgical, California, US.

The remaining authors declare that the research was conducted in the absence of any commercial or financial relationships that could be construed as a potential conflict of interest.

## Publisher’s note

All claims expressed in this article are solely those of the authors and do not necessarily represent those of their affiliated organizations, or those of the publisher, the editors and the reviewers. Any product that may be evaluated in this article, or claim that may be made by its manufacturer, is not guaranteed or endorsed by the publisher.
